# Antibacterial Activity of Two Metabolites Isolated From Endophytic Bacteria *Bacillus velezensis* Ea73 in *Ageratina adenophora*

**DOI:** 10.3389/fmicb.2022.860009

**Published:** 2022-05-06

**Authors:** Zhihua Ren, Lei Xie, Samuel Kumi Okyere, Juan Wen, Yinan Ran, Xiang Nong, Yanchun Hu

**Affiliations:** ^1^Key Laboratory of Animal Disease and Human Health of Sichuan Province, College of Veterinary Medicine, Sichuan Agricultural University, Yaan, China; ^2^College of Life Science, Leshan Normal University, Leshan, China

**Keywords:** *Ageratina adenophora*, endophytes, *Bacillus velezensis* Ea73, secondary metabolites, antibacterial compound

## Abstract

*Ageratina adenophora*, as an invasive and poisonous weed, seriously affects the ecological diversity and development of animal husbandry. Weed management practitioners have reported that it is very difficult to control *A. adenophora* invasion. In recent years, many researchers have focused on harnessing the endophytes of the plant as a useful resource for the development of pharmacological products for human and animal use. This study was performed to identify endophytes with antibacterial properties from *A. adenophora*. Agar well diffusion method and 16S rRNA gene sequencing technique were used to screen and identify endophytes with antibacterial activity. The response surface methodology and prep- high-performance liquid chromatography were used to determine the optimizing fermentation conditions and isolate secondary metabolites, respectively. UV-visible spectroscopy, infrared spectroscopy, nuclear magnetic resonance, and high-resolution mass spectrum were used to determine the structures of the isolated metabolites. From the experiment, we isolated a strain of *Bacillus velezensis* Ea73 (GenBank no. MZ540895) with broad-spectrum antibacterial activity. We also observed that the zone of inhibition of *B. velezensis* Ea73 against *Staphylococcus aureus* was the largest when fermentation broth contained 6.55 g/L yeast extract, 6.61 g/L peptone, 20.00 g/L NaCl at broth conditions of 7.95 pH, 51.04 h harvest time, and a temperature of 27.97°C. Two antibacterial peptides, Cyclo (*L*-Pro-*L*-Val) and Cyclo (*L*-Leu-*L*-Pro), were successfully extracted from *B. velezensis* Ea73. These two peptides exhibited mild inhibition against *S. aureus* and *Escherichia coli*. Therefore, we isolated *B. velezensis* Ea73 with antibacterial activity from *A. adenophora*. Hence, its metabolites, Cyclo (*L*-Pro-*L*-Val) and Cyclo (*L*-Leu-*L*-Pro), could further be developed as a substitute for human and animal antibiotics.

## Introduction

The abuse of antibiotics over the past few years has led to the emergence of drug-resistant pathogens, resulting in serious health complications in humans and animals (Ding et al., 2016; Chernov et al., 2019). Due to this setback, the search for new and effective antibiotics from natural sources has become an alternative strategy over the past few decades. The main sources of natural antibiotics are animals and plants. However, due to challenges, such as the bulky nature, slow-growing, endangered species, and high cost, associated with the use of animal and plant resources in excessive development of antibiotics, microorganisms may serve as the best alternative for harnessing pharmacological bioactive compounds for the development of drugs for both human and animal use because they are easy to handle and cheap to produce (Wen et al., 2022).

Studies have showed that endophytes are ubiquitous in all kinds of plants and are rich in species (Hallmann et al., 2011). They produce many secondary metabolites with various biological activities such as antibacterial and antitumor. They also enhance plant resistance to diseases and insect pests (Strobel et al., 2004; Tosi et al., 2021). Therefore, endophytes are a perfect alternative for the identification and production of antibacterial compounds.

The invasive nature of *Ageratina adenophora* (Spreng). R. M. King et H. Rob. is attributed to the plant's strong adaptability and breeding ability (Kong et al., 2017). The plant secretes allelochemicals that inhibit the growth of other plants, thereby forming a single dominant community and eventually destroying the ecological structure (Mcgeoch et al., 2010). *A. adenophora* has caused serious health conditions in animals, which has resulted in huge economic losses in the agriculture, forestry, and animal husbandry sectors (He et al., 2016; Tripathi et al., 2018; Okyere et al., 2020, 2021a; Cui et al., 2021; Ren et al., 2021a,b). Over the past few years, various control measures and strategies were developed to reduce the spread of *A. adenophora*; however, these programs have not yielded good results, and therefore, the utilization of the plant's resources (such as plant parts, extracts, metabolites, and endophytes) for the benefit of mankind has been the research direction over the past few years (Wan et al., 2010; Okyere et al., 2021b, 2022). *A. adenophora* has shown many biological activities such as insecticidal (Samuel et al., 2014), antibacterial (Yao et al., 2019), antitumor (Liao et al., 2014), antivirus (Jin et al., 2014), and antioxidation (Zhang et al., 2013).

Microorganisms have a complex symbiotic relationship with plants (Trivedi et al., 2020). Numerous studies have reported that endophytes produce similar secondary metabolites just as their host plant (Tanapichatsakul et al., 2018, 2019). Therefore, we hypothesized that endophytes from *A. adenophora* may possess antimicrobial activity, thus requiring investigation.

*Ageratina adenophora* endophytes have showed various biological activities. For example, a study by Jiang (2010) revealed that *Bacillus megaterium* isolated from *A. adenophora* showed growth-promoting activity (Jiang, 2010). Another study also reported that *Arbuscular mycorrhizal* fungi from *A. adenophora* had heavy metal repair activity (Kang, 2010). Fungi endophyte (*Coniochaeta* sp. F-8) also showed antioxidant activity (Fu et al., 2021). However, studies on the isolation of antibacterial endophytes and their metabolites from *A. adenophora* are limited; therefore, this study was performed to isolate antibacterial bacteria endophytes and their major metabolites from *A. adenophora*. This study will help us identify bacterial endophytes with antibacterial properties from *A. adenophora* to help in the development of probiotics and antibacterial drugs.

## Materials and Methods

### Plant Sample, Chemicals, and Reagents

Leaves, roots, stems, and flowers of *A. adenophora* were collected from Wangsuo Village, Cangzhou Street, Dechang County, Liangshan Yi Autonomous Prefecture, Sichuan Province (102°15′20″ E and 27°20′11″ N; elevation = 2,152 m). The samples were confirmed as *A. adenophora* by Prof. Chao Hu, Department of Botany, Sichuan Agricultural University. Culture media were purchased from Qingdao Hope Bio-Technology Co., Ltd., Qingdao, China. *Escherichia coli* ATCC 35218, *Salmonella tropina* H9812, *Pseudomonas aeruginosa* ATCC 27853, *Klebsiella pneumoniae* CMCC 46109, and *Staphylococcus aureus* CPCC 140594 were obtained from the College of Veterinary Medicine (Professor Xueqin Ni's lab), Sichuan Agricultural University, China.

### Isolation of Endophytic Bacteria

The fresh root, stem, leaf, and flower tissues of *A. adenophora* were washed with sterile water, then soaked in 3% NaClO for 3 min, 0.1% HgCl_2_ for 3 min, 75% ethanol for 3 min. Afterward, they were washed with sterile water five times and dried with sterile filter paper (Liang et al., 2009). The tissues were cut into 1 cm × 1 cm pieces with a sterile knife and plated on LB agar medium. After 2–3 days of culture in an incubator at 30 ± 2°C, the growing colonies were isolated and purified by cross-streaking on LB agar plates until pure strains were obtained.

### Screening of Antibacterial Activity

The indicator microorganisms used for antimicrobial activity assays throughout this study were *E. coli* ATCC 35218, *S. tropina* H9812, *P. aeruginosa* ATCC 27853, *K. pneumoniae* CMCC 46109, and *S. aureus* CPCC 140594. The indicator microorganisms were inoculated in LB broth and cultured at 37 ± 2°C in a 150 r/min shaker until it reached a logarithmic phase of about 14 h. After centrifugation at 4,000 r/min, the bacterial precipitates were collected and adjusted to a concentration of 1 × 10^6^ CFU/ml with sterile normal saline to form the pathogen suspension. The endophytic bacteria with antibacterial activity were screened by well diffusion method (du Toit and Rautenbach, 2000). Then, 60 μl sterile fermentation liquid was loaded into each well. Sterile normal saline was used as blank control. After incubation at 30 ± 2°C for 24 h, the inhibitory zone diameter (mm) was measured using a Vernier caliper. The experiment was repeated three times.

### Identification of Endophytic Bacteria

#### Morphological Identification

Strain Ea73 was inoculated on LB agar and cultured at 37 ± 2°C for 24 h. Morphological characteristics of the colony were observed using microscopy, and Gram staining was performed using Gram Stain Kit (Haibo Biotechnology Co., Ltd., Qingdao).

#### Molecular Identification

Endophytic bacterial isolate that exhibited antimicrobial activity was identified based on 16S rRNA sequence. The primers used to amplify the 16S rRNA sequence of the strain were 27F: 5′-AGAGTTTGATCCTGGCTCAG-3′ and 1492R: 5′-GCTTACCTTGTTACGACTT-3′. PCR amplification system (25 μl): template DNA 2 μl, 2 × Taq PCR Master Mix 12.5 μl, primer 27F (10 μmol/L) 1 μl, primer 1492R (10 μmol/l) 1 μl, DD H_2_O 8.5 μl. PCR reaction conditions: 95°C for 10 min. There were 35 cycles at 95°C for 1 min, 56°C for 30 s, 72°C for 2 min, 72°C for 10 min (Amin et al., 2020). The amplified products were sent to Youkang Biological (Chengdu) Co., Ltd. for sequencing and splicing. Consensus sequences were analyzed using BLASTN available from the National Center of Biotechnology Information (NCBI) website. Identity of endophytic bacteria was based on the percentage of homology to sequences available in the database. Further, MEGA 6 software was used to construct the phylogenetic trees using neighbor-joining and maximum parsimony method based on bootstrap values (1,000 replications) (Dereeper et al., 2010). The 16S rRNA gene sequence (1,497 bp) was submitted to NCBI GenBank with accession id MZ540895.

### Fermentation Condition Optimization

#### Optimization of Medium Composition

Taking the inhibitory zone diameter for *S. aureus* as an indicator, single-factor test was used to select the best carbon source, nitrogen source, inorganic salt, and their optimal concentration. The procedures are as follows.

##### Optimization of Carbon Source

The carbon source in LB medium was replaced with yeast extract, glucose, soluble starch, sucrose, and mannitol, and the best carbon source was selected under the same conditions. The optimal carbon source concentration was determined by adding different concentration gradients of 2.0, 5.0, 10.0, 15.0, 20.0, and 25.0 g/L, respectively.

##### Optimization of Nitrogen Sources

The nitrogen source in LB medium was replaced with tryptone, peptone, beef extract, (NH_4_)_2_SO_4_, and urea, and the best nitrogen source was selected under the same conditions. The optimal nitrogen source concentration was determined by adding different concentration gradients of 5.0, 10.0, 15.0, 20.0, 25.0, and 30.0 g/L, respectively.

##### Optimization of Inorganic Sources

The inorganic salt in LB medium was replaced with NaCl, MgSO_4_, KCl, CuSO_4_, and CaCl_2_, and the best inorganic salt source was selected under the same conditions. The optimal inorganic salt concentration was determined by adding different concentration gradients of 5.0, 10.0, 15.0, 20.0, 25.0, and 30.0 g/L, respectively.

According to the single-factor results, a Box-Behnken central composite design principle with carbon source (A), nitrogen source (B), and inorganic salt (C) as independent variables, and inhibitory zone diameter as response value, was adopted for carrying out a three-factor and three-level response surface test design. The results were statistically analyzed using Design Expert 7.0 statistical software (Sun et al., 2009).

#### Optimization of Fermentation Parameters

Taking the inhibitory zone diameter for *S. aureus* as an indicator, single-factor test was used to select the best fermentation initial pH, temperature, and time of medium. The procedures were as follows.

##### Fermentation pH

On the basis of optimal medium composition, pH was adjusted to 4.0, 5.0, 6.0, 7.0, 8.0, 9.0, and 10.0, respectively, and fermentation at 30°C for 48 h, to determine the best initial pH.

##### Fermentation Temperature

On the basis of optimal medium composition, pH was adjusted to 7. Fermentation was conducted at 24, 27, 30, 33, 36, and 39°C for 48 h to determine the optimal fermentation temperature.

##### Fermentation Time

On the basis of optimal medium composition, pH was adjusted to 7 and temperature to 30°C. Fermentation time of 24, 48, 72, 96, and 120 h, respectively, was used to determine the best fermentation time.

According to single-factor results, a Box-Behnken central composite design principle with initial pH (A), temperature (B), and time (C) as independent variables, and the inhibitory zone diameter as the response value, was adopted. Design Expert 7.0 statistical software was used to analyze the experimental results, and the optimal fermentation parameters were obtained (Sun et al., 2009).

### Isolation and Identification of Antibacterial Metabolites

Ea73 strain was activated and fermented under the optimum fermentation conditions. After the fermentation, the broth was broken by ultrasonic wave (40 Hz, 20 min), and the organic phase was collected by multiple extraction with ethyl acetate, and concentrated to dry at 45°C with rotary evaporator. The extractum was subjected to silica gel column chromatography with stepwise elution of chloroform:methanol (30:1, 20:1, 10:1, 6:1, 1:1; v/v). All eluted fractions were separately collected and concentrated using an evaporator. The fraction (20:1) was separated by gel filtration on Sephadex LH-20 column using running phase of 100% methanol. The eluted components were further purified by prep-high-performance liquid chromatography (HPLC) (Agilent 1260 series HPLC system):preparative reversed-phase column (10 μm, 250 mm × 20 mm), at a flow rate of 10 ml/min, methanol:H_2_O (25: 75, v/v), and UV detection at 210 nm. The purity of the separated compounds was further detected by HPLC (Agilent 1260 series HPLC system):C18 column (5 μm, 4.6 × 150 mm) at a flow rate of 1.0 ml/min, methanol-water (20–100% methanol in water over 8.0 min followed by 100% methanol to 13.0 min), and UV detection at 210 nm.

### Structural Identification

The structures of the two compounds were characterized using UV, infrared (IR), nuclear magnetic resonance (NMR), and high-resolution mass spectrum (HRMS). UV-visible spectrophotometer (AOI Instrument Co. Ltd., A390, Shanghai, China) was used to measure the UV-Vis spectra of the systems in this work. The Fourier-transform infrared spectroscopy (FTIR) spectrum, obtained from a Fourier transform infrared spectrometer (FTIR-840OS, Shimadzu, Japan), was used to identify functional groups. The structure of the compounds was determined using NMR spectroscopy (Bruker DRX 500 NMR instrument, Bruker, Rheinstetten, Germany) equipped with a 2.5 mm microprobe. NMR spectrometer using CDCl_3_ was deployed to measure ^1^H and ^13^C NMR. HRMS was performed on a Thermo Scientific Exactive Orbitrap LC-Mass Spectrometer with an electrospray ionization mode.

### Antibacterial Assay

Minimum inhibitory concentration (MIC) evaluation of compounds (**1** and **2**) was carried out in a 96-well plate according to the standard microdilution method. *E. coli* and *S. aureus* were used for this assay. The sterilized 96-well plates were taken from the 1st to the 11th rows from left to right, and 100 μl of sterilized MH medium was loaded into each well. This was done in triplicate. Afterward, 100 μl of the tested compound in the adjusted concentration was loaded into the second well. Then, it was continuously diluted to the 11th well. Afterward, 100 μl of mixture in the 11th well was discarded. Finally, 25 μl of the tested strain suspension was added to make the final concentration of the compound 512, 256, 128, 64, 32, 16, 8, 4, 2, and 1 μg/ml. Both blank control and positive control had no tested compound. Using the obvious growth of the negative control wells as an indicator, the sterile growth and non-turbidity holes in the 96-well plate were considered the MIC holes (Jeong et al., 2017).

### Statistical Analysis

Statistical analysis of the data collected (from various independent experiments) was performed using SPSS 22 Statistical Analysis Software (SPSS Inc., Chicago, IL, USA). All experimental results are presented as mean ± SD, and statistical significance was determined by one-way analysis of variance (ANOVA) followed by Tukey's test. The values were significantly different at *p* < 0.05.

## Results

### Screening of Strain Ea73

Out of the 95 endophytic bacteria that were isolated from the roots, stems, leaves, and flowers of *A. adenophora*, only 21 strains showed antibacterial activity after screening using the well diffusion method. These 21 strains had inhibitory effect on one or more pathogenic bacteria. However, strain Ea73 had universal antibacterial activity against all the five tested pathogenic bacteria used in this study with *S. aureus* being the most inhibited pathogenic bacteria (Table 1). Strain Ea73 was submitted for preservation in the China Center for Typical Culture Collection (CCTCC) on September 6, 2021, preservation number CCTCC M 20211139.

**Table 1:** Growth inhibitory zone diameter (mm) of pathogens with Ea73 bacteria isolated from *Ageratina adenophora*.

**Pathogens**	**Inhibitory zone diameter (mm)**
*Escherichia coli* ATCC 35218	10.57 ± 0.18
*Salmonella tropina* H9812	9.69 ± 0.17
*Pseudomonas aeruginosa* ATCC 27853	13.72 ± 0.36
*Klebsiella pneumoniae* CMCC 46109	11.00 ± 0.09
*Staphylococcus aureus* CPCC 140594	32.16 ± 2.04

### Identification of Endophytic Bacteria

**Figure 1:**
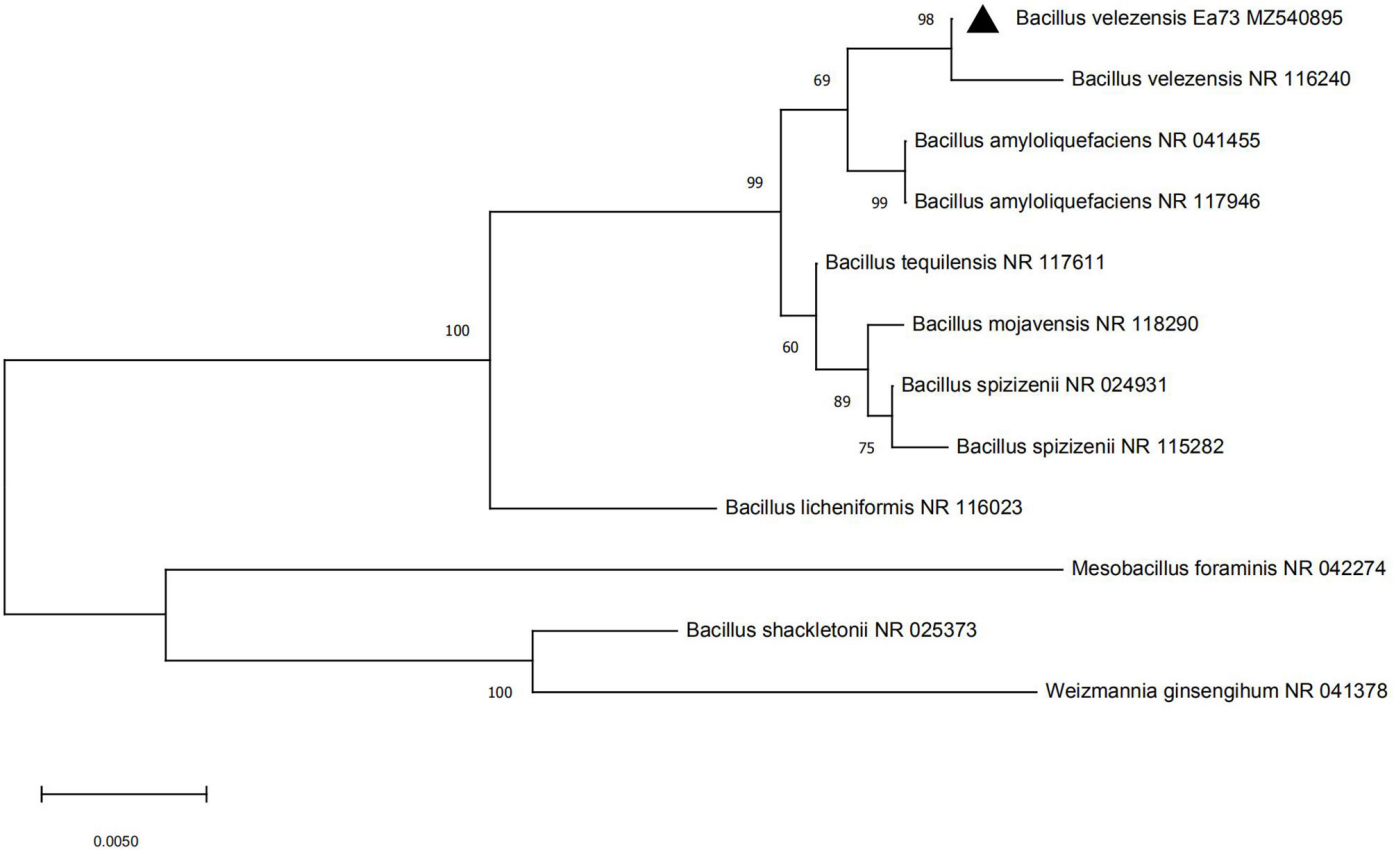
Phylogenetic tree of *Bacillus velezensis* strain Ea73.

Strain Ea73 was a short rod-shaped and Gram-positive bacteria. It also showed colony morphology characteristics of milky white, round or oval, opaque, rough surface, neat edges, and smooth myxoid colonies. The 16S rDNA sequence of strain Ea73 was analyzed using BLAST with a known nucleic acid sequence in GenBank. The results showed that strain Ea73 was similar to *Bacillus*. In addition, phylogenetic analysis results showed that the strain was in the same minimum branch as *Bacillus velezensis*, whose accession number was NR116240 (Figure 1). Therefore, strain Ea73 was identified as *B. velezensis* and named as *B. velezensis* Ea73. The gene sequence was submitted to GenBank with accessory number MZ540895.

### Fermentation Condition Optimization

#### Optimizing the Medium Composition by Response Surface Methodology

**Figure 2:**
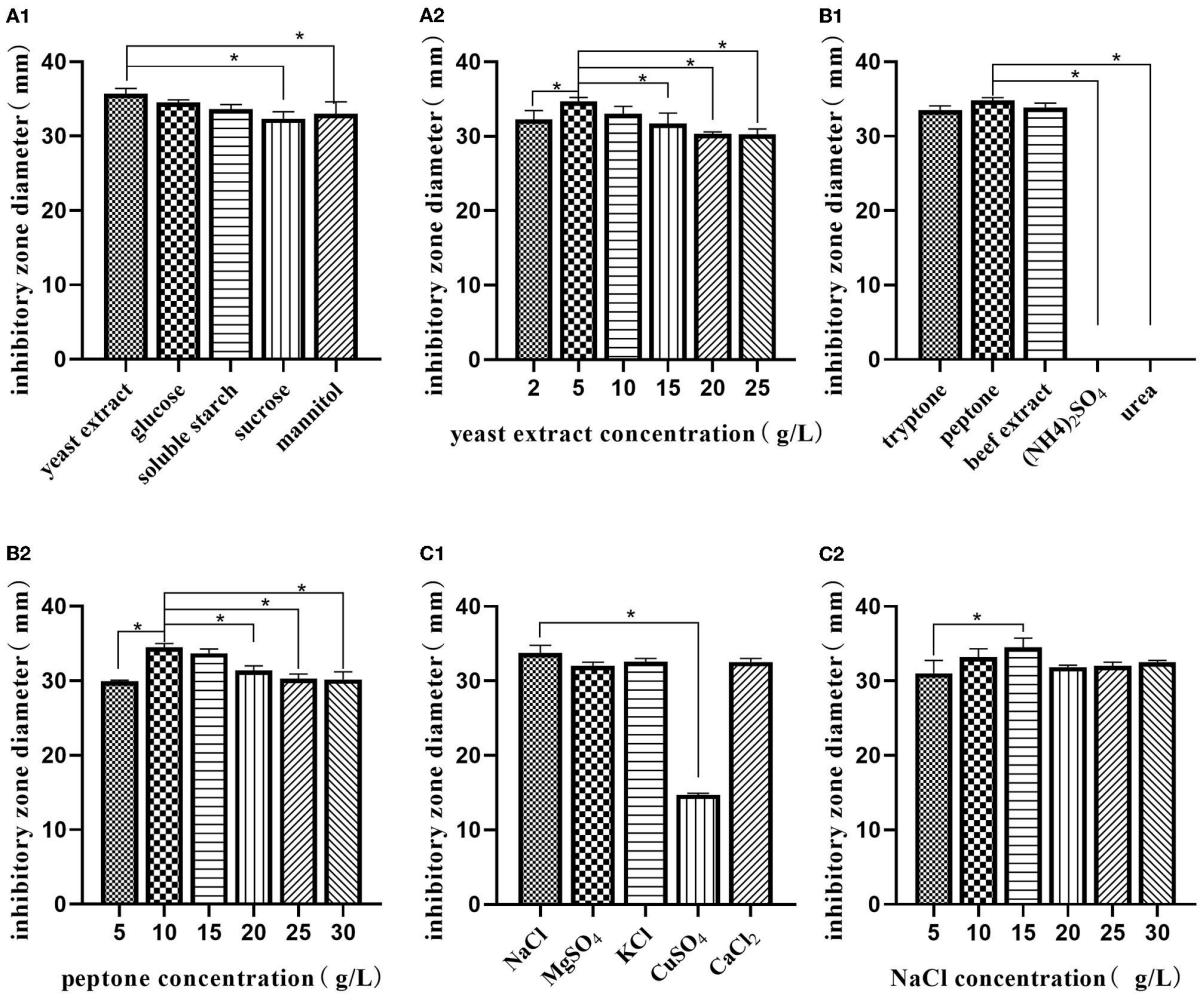
Single-factor analysis of medium composition. Effect of various sources of carbon **(A1)**; effects of different concentrations of yeast extract **(A2)**; effect of various sources of nitrogen **(B1)**; effects of different concentrations of peptone **(B2)**; effect of various sources of inorganic salt **(C1)**; effects of different concentrations of NaCl **(C2)**. The values are presented as the mean ± SD. Bars with * differed significantly (*n* = 3, *p* < 0.05).

Using a single-factor experiment, we observed that the best carbon source was yeast extract (5.0 g/L), nitrogen source was peptone (10.0 g/L), and the best inorganic salt was NaCl (15.0 g/L) (Figure 2). A response surface design was further applied when the optimal region for running the process was being identified. Taking carbon source (A), nitrogen source (B), and inorganic salt (C) as independent variables and the inhibitory zone diameter as response values, a three-factor and three-level response surface experiment was designed (Table 2).

**Table 2:** Design and experimental results of Box–Behnken design (medium composition).

**Run**	**A (g/L)**	**B (g/L)**	**C (g/L)**	**Response (mm)**
1	2	5	15	32.14
2	10	5	15	33.39
3	2	15	15	30.58
4	10	15	15	32.64
5	2	10	10	32.32
6	10	10	10	33.61
7	2	10	20	33.45
8	10	10	20	33.65
9	5	5	10	32.91
10	5	15	10	33.68
11	5	5	20	34.15
12	5	15	20	31.60
13	5	10	15	34.12
14	5	10	15	33.93
15	5	10	15	33.73

The Design Expert software was used to perform quadratic multiple regression fitting for the experimental data (Table 3). The quadratic multiple regression model equation of R for each factor was represented as R = 34.14 + 0.60 A-0.49 B + 0.016 C + 0.18 AB-0.20 AC-0.83 BC-1.00 A^2^-0.96 B^2^ + 0.11 C^2^. The regression model was significant (*p* < 0.05), indicating that the experimental model was statistically significant. According to the *p*-value, the variables A, B, BC, A^2^, and B^2^ in the model had a significant effect on the inhibitory zone diameter (*p* < 0.05), indicating that there is no linear relationship between the experimental factors and the response value; however, the interaction of the primary term, quadratic term, and BC had a close relationship with the response value. Lack of fit was not significant (*p* > 0.05), indicating that the fitting degree of the experimental model was good, the selection of the model was reasonable, and the residual of the model may be a result of the random error in the experimental process. The correction determination coefficient $R_{Adj}^{2}$ = 0.7738 of the model shows that 77.38% of the variation in the experiment was distributed in the factors of the equation, and the *R*^2^-value is 0.9192, indicating that there is a good fit between the measured value and the predicted value of inhibitory zone diameter. The model can be used to predict the actual situation of inhibitory zone diameter.

**Table 3:** ANOVA results of the quadratic model (medium composition).

**Source**	**Sum of squares**	**df**	**Mean square**	***F*-Value**	***p*-Value, Probability > F**
Model	13.50	9	1.50	6.32	0.0281
A	2.88	1	2.88	12.14	0.0176
B	1.85	1	1.85	7.81	0.0382
C	1.947E-003	1	1.947E-003	8.202E-003	0.9314
AB	0.13	1	0.13	0.57	0.4861
AC	0.17	1	0.17	0.72	0.4353
BC	2.76	1	2.76	11.61	0.0191
A2	3.13	1	3.13	13.17	0.0151
B2	3.37	1	3.37	14.21	0.0130
C2	0.048	1	0.048	0.20	0.6713
Residual	1.19	5	0.24		
Lack of fit	1.11	3	0.37	9.73	0.0946
Pure error	0.076	2	0.038		
Cor total	14.69	14			

**Figure 3:**
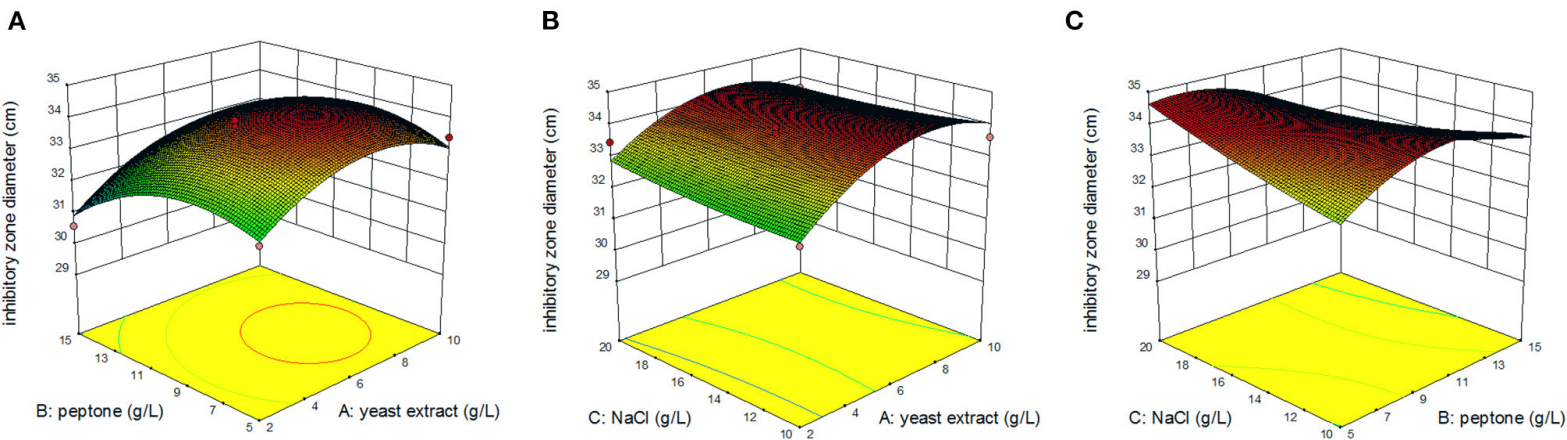
Response surface of inhibition zone under the interaction of peptone and yeast extract concentration **(A)**; yeast extract and NaCl concentration **(B)**; peptone and NaCl concentration **(C)**.

Figure 3 displays the response surface curves as interaction between the yeast extract, peptone, and NaCl on inhibitory zone diameter. It directly shows the response over a region of independent variables and the relationship between experimental levels of each factor. Each figure demonstrates the effect of two factors while the other factor was fixed at zero level. Figure 3A represents the effect of yeast extract concentration and peptone concentration on inhibitory zone diameter at a fixed NaCl concentration of 15.0 g/L. The results showed that the maximum response value was obtained when the yeast extract concentration was 7.0 g/L and peptone concentration was 9.0 g/L. The effect of yeast extract concentration and NaCl concentration on the inhibitory zone diameter at a fixed peptone concentration of 10.0 g/L is shown in Figure 3B. Decreasing the yeast extract concentration led to an increase in the inhibitory zone diameter, irrespective of the NaCl concentration. The response value that reached its highest point at yeast extract concentration was 7.0 g/L. Figure 3C shows the effect of peptone concentration and NaCl concentration on the inhibitory zone diameter at a fixed yeast extract concentration of 5.0 g/L. Increasing the concentration of NaCl resulted in an increase in the response surface. The response value reached its highest point at 7.0 g/L of peptone concentration and 20.0 g/L of NaCl concentration. Therefore, from the surface response graphs and the regression analysis of the equation, it was concluded that the optimal conditions for inhibitory zone diameter were located in the region where yeast extract, peptone, and NaCl concentration were 6.55, 6.61, and 20.00 g/L, respectively.

#### Optimizing the Fermentation Parameters by Response Surface Methodology

**Figure 4:**
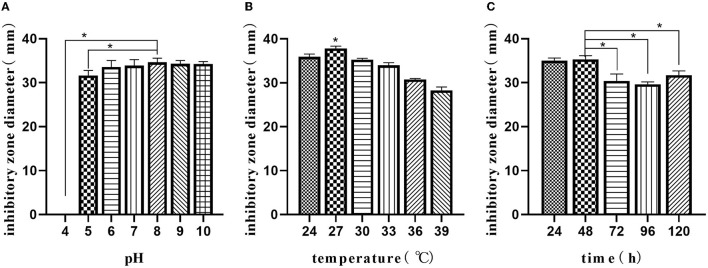
Single-factor analysis of fermentation parameters. Effect of initial pH **(A)**; effects of temperature **(B)**; effect of time **(C)**. The values are presented as the mean ± SD. Bars with * differed significantly (*n* = 3, *p* < 0.05).

The optimal initial pH, temperature, and time were selected using the composition of media in the “Optimizing the medium composition by response surface methodology” section. Using single-factor screening, we observed that the initial pH, temperature, and time of the center were 8, 27°C, and 48 h, respectively (Figure 4). Taking initial pH (A), temperature (B), and time (C) as independent variables and inhibitory zone diameter as response values, a three-factor and three-level response surface experiment was designed (Table 4).

**Table 4:** Design and experimental results of Box–Behnken design (fermentation parameters).

**Run**	**A**	**B (°C)**	**C (h)**	**Response (mm)**
1	7	24	48	36.34
2	9	24	48	36.30
3	7	30	48	38.93
4	9	30	48	37.73
5	7	27	24	35.88
6	9	27	24	36.45
7	7	27	72	36.18
8	9	27	72	36.77
9	8	24	24	30.81
10	8	30	24	35.95
11	8	24	72	36.00
12	8	30	72	38.27
13	8	27	48	40.25
14	8	27	48	40.16
15	8	27	48	40.99

The Design Expert software was used to perform quadratic multiple regression fitting for the experimental data (Table 5). The quadratic multiple regression model equation of R for each factor was represented as *R* = 40.47-0.010 A + 1.43 B + 1.02 C-0.29 AB + (5.000E-003) AC-0.72 BC-1.04 A^2^-2.10 B^2^-3.11 C^2^. The regression model was significant (*p* < 0.05), and the variables B, C, B^2^, and C^2^ in the model had a significant effect on the inhibitory zone diameter (*p* < 0.05). Lack of fit was not significant (*p* > 0.05). The correction determination coefficient was $R_{Adj}^{2}$ = 0.7221 and the *R*^2^-value was 0.9192, indicating that the model could be used to predict the actual situation of inhibitory zone diameter.

**Table 5:** ANOVA results of the quadratic model (fermentation parameters).

**Source**	**Sum of squares**	**df**	**Mean square**	***F*-Value**	***p*-Value, Probability > F**
Model	77.19	9	8.58	5.04	0.0448
A-pH	8.000E-004	1	8.000E-004	4.703E-004	0.9835
B-temperature	16.33	1	16.33	9.60	0.0269
C-time	8.26	1	8.26	4.86	0.0787
AB	0.34	1	0.34	0.20	0.6751
AC	1.000E-004	1	1.000E-004	5.879E-005	0.9942
BC	2.06	1	2.06	1.21	0.3214
A^2^	3.99	1	3.99	2.35	0.1862
B^2^	16.32	1	16.32	9.59	0.0269
C^2^	35.65	1	35.65	20.95	0.0060
Residual	8.51	5	1.70		
Lack of fit	8.09	3	2.70	13.00	0.0723
Pure error	0.41	2	0.21		
Cor total	85.70	14			

**Figure 5:**
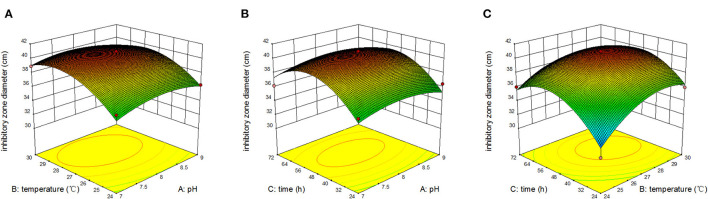
Response surface of inhibition zone under the interaction of pH and temperature **(A)**; pH and time **(B)**; temperature and time **(C)**.

Figure 5 displays the response surface curves from the interaction among initial pH, temperature, and time on inhibitory zone diameter. Figure 5A represents the effect of original pH and temperature on zone of inhibition at a fixed time of 48 h. The results showed that the maximum response value was obtained when pH was 8 and temperature was 28°C. Figure 5B also represents the effect of original pH and time on inhibitory zone diameter at a fixed temperature of 27°C. The result showed that the maximum response value was obtained when the original pH was 8 and time was 51 h. Figure 5C represents the effect of original pH and time on inhibitory zone diameter at a fixed pH of 8. We observed that the maximum response value was obtained when the temperature was 28°C and time was 51 h. Therefore, from the surface response graphs and the regression analysis of the equation, it was concluded that the optimal conditions for inhibitory zone diameter were located in the region where initial pH, temperature, and time were 7.95, 27.97°C, and 51.04 h, respectively.

In conclusion, the results showed that the yield of antibacterial metabolites reached the maximum when the yeast extract was 6.55 g/L, peptone was 6.61 g/L, NaCl was 20.00 g/L, initial pH was 7.95, time was 51.04 h, and temperature was 27.97°C. Under these conditions, the inhibitory zone diameter of Ea73 fermentation broth against *S. aureus* reached 40.76 mm.

### Isolation and Identification of Antibacterial Metabolites From *B. velezensis* Ea73

**Figure 6:**
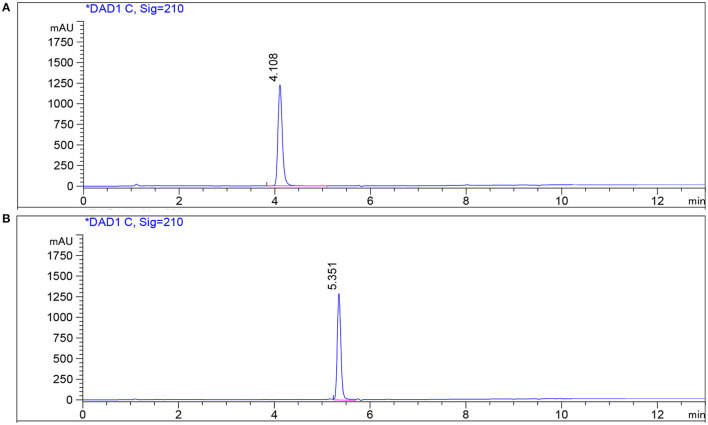
HPLC profile of compounds on a reversed-phase C18 HPLC column. **(A)** Standard and compound **1** in fermentation broth; **(B)** standard and compound **2** in fermentation broth.

Using ethyl acetate, 4 L of fermentation broth was extracted, concentrated, and dried to obtain about 1 mg crude extract. The crude extract was further separated into five components (Fr_1_-Fr_5_) by silica gel column chromatography. After antibacterial activity and HPLC detection, the Fr_4_ components at 20:1 (chloroform/methanol) elution concentration were further separated into Fr_4−1_ and Fr_4−2_ by Sephadex LH-20 column. The two components were purified by the prep-HPLC, respectively. Compound **1** (3.4 mg) was obtained by collecting components at the retention time 4.19 min of Fr_4−1_. Compound **2** (5 mg) was obtained by collecting components at the retention time 4.58 min of Fr_4−2_. The purity of the two compounds was detected by HPLC. The purity of the compounds recorded was more than 98%, according to the peak area from the chromatogram (Figure 6).

### Characterization of Compound

**Figure 7:**
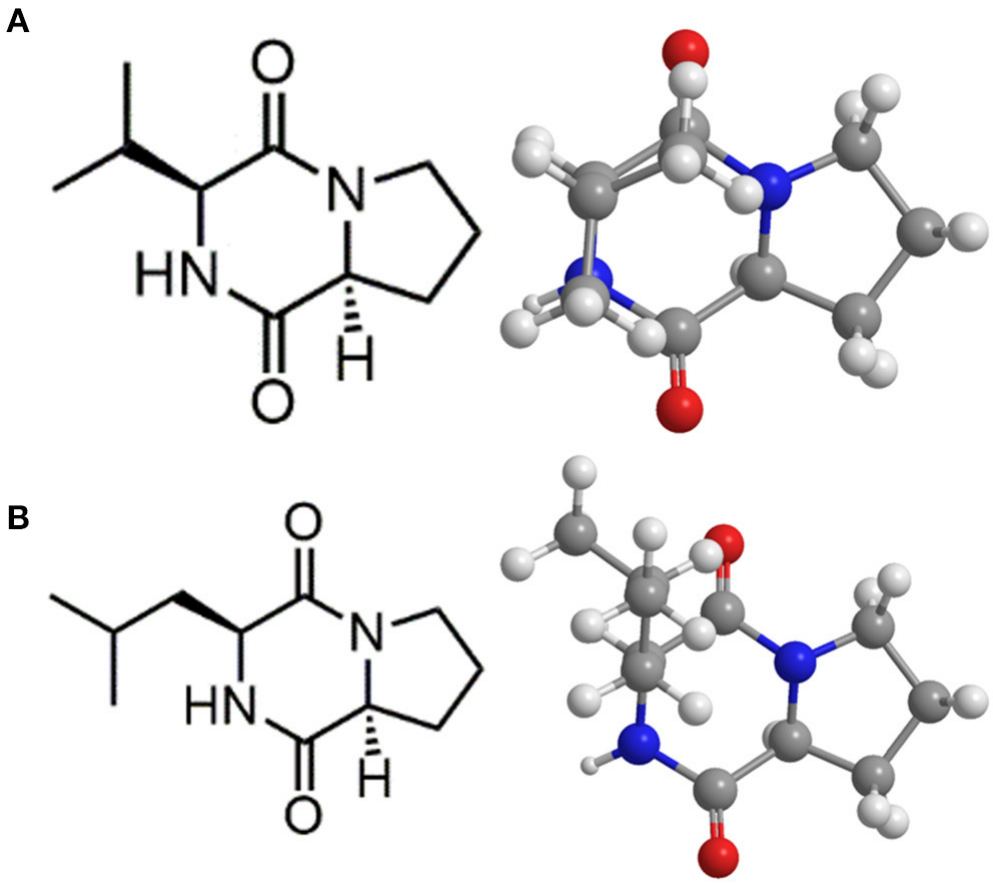
Chemical structure of cyclic dipeptides. Cyclo (*L*-Pro-*L*-Val) **(A)**; Cyclo (*L*-Leu-*L*-Pro) **(B)**.

Compound **1** was isolated as a white powder. The molecular formula of compound **1** was established as C_10_H_16_N_2_O_2_ by HRESIMS at *m/z* 197.1284 [M+H]^+^ (calculated for C_10_H_16_N_2_O_2_: 197.1282). The IR spectrum showed the characteristic absorption at 3,438 cm^−1^ and 1,656 cm^−1^, suggestive of imino group and carbonyl group, respectively. In the UV spectrum, there was no obvious ultraviolet absorption. ^1^H-NMR spectra showed that the active hydrogen signal in one amide was δ 6.07 (1H, s), and four hydrogen were between δ4.2 and 3.5, adjacent to the regions of nitrogen, in which 4.09–4.05 (m, 1H), and 3.95–3.90 (m, 1H) were simultaneously absorbed by carbonyl and nitrogen with a larger chemical shift; 3.67–3.59 (m, 1H), and 3.57–3.48 (m, 1H) were hydrogen on chiral carbon, and two double peaks near δ 1.0, 1.06 (d, J = 7.3 Hz, 3H), 0.90 (d, J = 6.8 Hz, 3H) belong to methyl hydrogen. ^13^C NMR carbon spectrum and DEPT spectrum give 10 carbon signals, including 2 methyl signals δ 19.23, 16.07; 3 methylene signals δ 45.15, 28.54, 22.38; and 3 methylene signals δ 60.40, 58.83, 28.39. Among them, δ 170.08, 164.94 was the carbonyl peak, and δ 60–40 was the leading carbon of nitrogen (Supplement Figure 1, Table 6). By comparing the above information with the literature (Pedras et al., 2005), compound **1** was determined to be (3S,8aS)-3-propan-2-yl-2,3,6,7,8,8a-hexahydropyrrolo[1,2-a]pyrazine-1,4-dione, also known as Cyclo (*L*-Pro-*L*-Val) (Figure 7A).

**Table 6:** ^1^H and ^13^C NMR data for compounds **1** and **2** (CDCl_3_, 500 MHz, δ in ppm).

**Positions**	**Compound 1**		**Compound 2**	
	**δ^C(ppm)^**	**δ^H^, Mult. (J in Hz)**	**δ^C(ppm)^**	**δ^H^, Mult. (J in Hz)**
1	170.08		170.08	
NH		6.07 (s, 1H)		5.77 (s, 1H)
2	60.40	3.95–3.90 (m, 1H)	59.00	4.02 (dd, *J* = 9.5, 3.7 Hz, 1H)
3	164.94		164.94	
4	45.15	3.67–3.59 (m, 1H)	45.53	3.65–3.51 (m, 2H)
		3.57–3.48 (m, 1H)		
5	28.54	2.07–1.99 (m, 2H)	28.14	2.10–1.98 (m, 2H)
6	22.38	2.40–2.33 (m, 1H)	22.76	2.17–2.10 (m, 1H)
		1.96–1.84 (m, 1H)		1.97–1.83 (m, 1H)
7	58.83	4.09–4.05 (m, 1H)	53.38	4.15–4.08 (m, 1H)
8	28.39	δ 2.69–2.56 (m, 1H)	24.75	2.40–2.30 (m, 1H)
9	19.23	1.06 (d, *J* = 7.3 Hz, 3H)	23.32	1.06 (d, *J* = 7.3 Hz, 3H)
10	16.07	0.90 (d, *J* = 6.8 Hz, 3H)	21.18	0.90 (d, *J* = 6.8 Hz, 3H)
11			38.64	1.79–1.66 (m, 1H)
				1.52 (ddd, *J* = 14.5, 9.6, 5.0 Hz, 1H)

Compound **2** was isolated as a white powder. The molecular formula of **2** was established as C_11_H_18_N_2_O_2_ by HRESIMS at *m/z* 211.1441 [M + H]^+^ (calculated for C_11_H_18_N_2_O_2_: 211.1438). The IR spectrum showed characteristic absorption at 3,430 and 1,660 cm^−1^, suggestive of imino group and carbonyl group, respectively. In the UV spectrum, there was no obvious ultraviolet absorption. The NMR spectrum of compound **2** was very similar to that of compound **1**, with one more methylene (δ38.64) (Supplement Figure 2, Table 6). Based on NMR data and literature analysis (Pedras et al., 2005), compound **2** was determined to be (3S,8aS)-3-(2-methylpropyl)-2,3,6,7,8,8a-hexahydropyrrolo[1,2-a]pyrazine-1,4-dione, also known as Cyclo (*L*-Leu-*L*-Pro) (Figure 7B).

### Antibacterial Assay of Two Compounds

The MIC values of the two compounds were determined using a 96-well plate assay. Both compounds were found to have inhibitory effects on *E. coli* ATCC 35218 and *S. aureus* CPCC 140594. Cyclo (*L*-Pro-*L*-Val) showed the MIC values of 512 and 256 μg/ml for *E. coli* and *S. aureus*, respectively, whereas Cyclo (*L*-Leu-*L*-Pro) showed an MIC value of 512 μg/ml for both two pathogenic bacteria.

## Discussion

In this study, *B. velezensis* was successfully isolated from *A. adenophora*, and its fermentation broth had general antibacterial activity against *E. coli, Salmonella, P. aeruginosa, K. pneumonia*, and *S. aureus*. *B. velezensis* have similar molecular characteristics to *Bacillus amyloliquefaciens* and *Bacillus methylotrophicus* (Rabbee et al., 2019). Due to its excellent biocontrol effect, *Bacillus spp*. have been useful in the field of agricultural production. Research on *B. velezensis* is mainly focused on promoting animal and plant growth, antagonizing pathogens, inducing systemic resistance, identifying bacteriostatic substances and their gene clusters, and antagonistic mechanism (Gao et al., 2017; Cao et al., 2018; Chen et al., 2018). In the prevention and control of plant diseases, *B. velezensis* has showed good antagonistic effect against many plant pathogenic fungi and bacteria, such as *Alternaria solani, Phytophthora capsici, Fusarium solani, Botrytis cinerea Pers, Fusarium oxysporum, Streptomyces galilaeus*, and *Rhizoctonia solani* (Ait Kaki et al., 2013; Kanjanamaneesathian et al., 2013; Lim et al., 2017). In animals, it has shown good inhibitory effect on common pathogens such as *E. coli, Salmonella enteritis, Campylobacter jejuni, Listeria monocytogenes, Aeromonas hydrophila, Streptococcus agalactis*, and *Vibrio parahaemolyticus* (Nannan et al., 2018; Yi et al., 2018). In this study, we observed that the fermentation broth of *B. velezensis* had good antibacterial effect, especially against *S. aureus*, with an inhibitory zone diameter of 32.16 ± 2.04 mm. Therefore, *B. velezensis* Ea73 is expected to be an important strain resource for the development of natural antibiotic products.

Fermentation broths do not only contain a small amount of metabolic active substances, but also a large number of impurities that affect the antimicrobial activity. The type and proportion of compounds in the culture medium affect the antimicrobial potency of the fermentation broth (Sanchez and Demain, 2002). Therefore, there is the need to investigate the ideal broth conditions that will yield the maximum antimicrobial activity. In this study, taking the inhibitory activity of fermentation broth against *S. aureus* as an index, a single-factor test combined with response surface analysis was used to optimize the proportion of culture medium and fermentation conditions of *B. velezensis* Ea73. The results showed that the inhibitory activity of strain Ea73 was affected in the order of yeast extract > temperature > peptone > time > NaCl > pH. In addition, inhibitory activities were effective when the yeast extract in the media was 6.55 g/L, peptone was 6.61 g/L, NaCl was 20.00 g/L, initial pH was 7.95, temperature was 27.97°C, and harvest time was 51.04 h. At these media conditions, the inhibitory zone diameter of strain Ea73 against *S. aureus* reached the maximum (which was 40.76 mm). Therefore, compared to the inhibitory zone diameter produced by fermentation broth before optimization, the antibacterial activity of strain Ea73 was significantly improved after optimization.

Studies have shown that *B. velezensis* has a remarkable ability to produce secondary metabolites with strong antimicrobial properties such as lipopeptides, surfactin, fengycin, and bacillomycin D, macrolactin, bacillaene, difficidin, or oxydifficidin; and peptides (Chen et al., 2015; Khalid et al., 2021b). However, the strain type, composition, and conditions of fermentation medium affect the yield and type of secondary antibacterial metabolites produced by various strains (Khan et al., 2017; Arokiyaraj et al., 2019). For example, Pournejati et al. (2019) optimized the fermentation conditions of *B. velezensis* RP137 and found that when rice starch and potassium nitrate were supplied to the strain RP137, it increased the production of aminoglycoside antibacterial metabolites. Khalid et al. (2021a) optimized the fermentation parameters (temperature, time, pH, and loaded liquid volume) for *B. velezensis* JTYP2 and found that all these parameters differently affected β-glucanase and protease production. The main antibacterial metabolites isolated by Nam and colleagues from *B. velezensis* NST6 was C15-bacillomycin D (Nam et al., 2021). Therefore, after optimizing the antibacterial activity of strain Ea73, we continued to isolate and purify its antibacterial metabolites.

The two antibacterial metabolites that were purified from the fermentation broth of *B. velezensis* Ea73 were identified as Cyclo (*L*-Pro-*L*-Val) and Cyclo (*L*-Leu-*L*-Pro) by NMR, IR, and HRMS. The above results were further confirmed by comparing the physical and spectral data of the two cyclic dipeptides (CDPs) with the values described in literatures. CDPs are formed by the internal cyclization of two amino acid amides, and a matrix of 2-diketo-piperazine or 2-dioxopiperazine (Saadouli et al., 2020). They are the simplest, naturally occurring cyclic forms of peptides, commonly biosynthesized by a large variety of living organisms and conserved in bacteria to humans (Bojarska et al., 2021).

Many studies have showed that CDPs possess a variety of biological properties such as antibacterial (Nishanth Kumar et al., 2012), antifungal (Ström et al., 2002), anticancer (Nishanth et al., 2014), neuroprotective (Bellezza et al., 2014), blood–brain barrier transporter (Teixidó et al., 2007), anticoagulation (Newman et al., 2003), anti-inflammatory (Minelli et al., 2012), and plant growth regulation activity (Ortiz-Castro et al., 2011). In this study, we observed that Cyclo (*L*-Pro-*L*-Val) and Cyclo (*L*-Leu-*L*-Pro) showed antibacterial effect on *S. aureus* and *E. coli*; however, it was not significant. This was similar to the results of Furtado et al. (2005), which isolated 7 CDPs [including Cyclo (*L*-Pro-*L*-Val) and Cyclo (*L*-Pro-*L*-Leu)] from *Aspergillus fumigatus* fermentation broth and reported that all the CDPs inhibited the growth of *S. aureus* and *Micrococcus luteus* at the concentration of 2.9 mmol/L. In other studies, the MIC value of Cyclo (*L*-Leu-*L*-Pro) against *L. monocytogenes* ATCC 19111 was found to be 512 μg/ml (Gowrishankar et al., 2016), whereas MIC value of Cyclo (Leu-Pro) on *P. aeruginosa* PAO1 was 250 μg/ml (Parasuraman et al., 2020). In contrast, Kaaniche et al. (2020) revealed that both Cyclo (Leu-Pro) and Cyclo (Val-Pro) demonstrated significant antibacterial activity against *Agrobacterium tumefaciens* ATCC 23308, *L. monocytogenes* ATCCC 19117, *S. aureus* ATCC 6538, and *Salmonella typhimurium* ATCC 14028, at MIC values of (10, 20, 20, 10) and (12, 20, 20, 10) μg/ml, respectively.

The differences in activity may be attributed to the absolute configuration. For example, a study by Kaaniche et al. (2020) showed that five CDPs [Cyclo (*D*-Phe-*D*-Pro), Cyclo (*D*-Leu-*D*-Pro), Cyclo (*D*-Pro-*D*-Val), Cyclo (*D*-Ile-*D*-Pro), and Cyclo (*D*-Phe-*trans*-4-OH-*D*-Pro)] with the *D*-configuration of the amino acid had different MIC values [Cyclo (*D*-Phe-*L*-Pro) had MIC value of 0.13 μg/ml, Cyclo (*D*-Phe-*D*-Pro) had MIC value of 0.03 μg/ml, and the diastereomer Cyclo (*L*-Phe-*D*-Pro) had MIC value of 0.10 μg/mL] against *Vibrio anguillarum*. Furthermore, comparing their stereoisomers, the authors pointed out that at least one *D*-amino acid was required for antibacterial activity. Although different combinations of *D*- and *L*-amino acids activate antibacterial activity, the DD-enantiomers have higher activity (Kaaniche et al., 2020). This was confirmed by Kumar et al. (2012), who reported that the MIC values of Cyclo (*L*-Leu-*L*-Pro) against *S. aureus* and *E. coli* were 32 and 250 μg/ml, respectively, whereas that of Cyclo (*D*-Pro-*L*-Leu) to the same bacteria were 64 and 32 μg/ml. These results indicated that, among the three enantiomers (*LL, DL*, and *DD*), the antibiotic activity of *DD*- enantiomers seems to be the highest, and the activity of *D*-amino acids is stronger than that of *L*-enantiomers. This confirms the report that *D*-amino acids are responsible for biofilm disassembly of *Bacillus subtilis* (Kolodkin-Gal et al., 2010; Hochbaum et al., 2011). However, the relationship between enantiomers and activity was not fully confirmed in this study, hence it needs further studies.

A large number of studies have shown that CDPs also have synergistic antibacterial effect. A study by Kumar et al. (2012) reported the synergistic activity of Cyclo (*L*-Pro-*L*-Leu), Cyclo (*D*-Pro-*L*-Leu), and Cyclo (*D*-Pro-*L*-Tyr) against bacteria *in vitro*. The combination of CDPs and amphotericin B or clotrimazole also had synergistic effect against *Candida albicans in vitro* (Kumar et al., 2013). Rhee (2004) studied the synergistic antibacterial effect of two CDPs Cyclo (*L*-Leu-*L*-Pro) and Cyclo (*L*-Phe-*L*-Pro) *in vitro*. The results showed that the combination of the two drugs could effectively inhibit the growth of vancomycin-resistant enterococci with MIC value of 0.25–1 mg/L. In addition, it also showed a strong inhibitory effect on pathogenic bacteria such as *E. coli, S. aureus*, and *C. albicans* at MIC value of 0.25–0.5 mg/L. Combination therapy can be used to expand the antimicrobial spectrum to prevent the emergence of resistant organisms. Therefore, the synergistic activity of CDPs can be developed and utilized to eliminate pathogens.

There is more to the development and utilization of CDPs as we can focus on the molecular self-assembly properties of CDPs. CDPs contains a six-membered cyclolactam ring with two amide bonds, in which functional groups could be presented at a total of six locations and four positions controlled by stereochemistry (Borthwick, 2012; Borgman et al., 2019). The diverse functional properties and applications arise from the unique structural attributes of CDPs, conformational rigidity, strong intermolecular interactions, proteolytic stability, biological relevance, and biocompatibility (Balachandra et al., 2021). Rationally designed CDPs have been utilized for the development of unique materials such as low-molecular-weight gelators (Kleinsmann and Nachtsheim, 2013), AIEgenic systems (Balachandra and Govindaraju, 2020), antioxidant CDPs (Manchineella et al., 2017), and quantum-confined materials (Tao et al., 2018). CDPs are also scaffold for the syntheses of various complex natural products (González et al., 2012). Overall, the structural and functional diversity of CDPs exemplify its significance and utility across the domains of chemistry, biology, and materials science.

In this study, we successfully isolated and characterized *B. velezensis* Ea73 from *A. adenophora. B. velezensis* Ea73 showed a strong antibacterial activity and genetic stability against *E. coli, Salmonella, P. aeruginosa, K. pneumonia*, and *S. aureus*. We also found that the maximum antibacterial activity was observed when the concentrations of yeast extract, peptone, and NaCl in the culture media were 6.55, 6.61, and 20.00 g/L, respectively, and the initial pH, harvesting time, and temperature of the culture media were 7.95, 51.04 h, and 27.97°C, respectively. Under these conditions, the inhibitory zone diameter of Ea73 fermentation broth against *S. aureus* reached 40.76 mm. Furthermore, two antibacterial peptides, Cyclo (*L*-Pro-*L*-Val) and Cyclo (*L*-Leu-*L*-Pro), were successfully isolated from *B. velezensis* Ea73. These two CDPs had mild antibacterial activity against *S. aureus* and *E. coli*. Therefore, we stipulated that *A. adenophora* contains antibacterial endophytes with numerous antibacterial metabolites that may serve as alternative sources for the development of natural antibiotics; however, further studies are still required to elucidate the complete mechanisms of action by confirming the efficacy of these two compounds through *in vivo* experiments.

## Data Availability Statement

The original contributions presented in the study are included in the article/Supplementary Material, further inquiries can be directed to the corresponding author/s.

## Author Contributions

ZR, LX, and SO: conceptualization, methodology, and software. ZR, LX, SO, JW, and YR: data collection, writing, and original draft preparation. SO, JW, XN, and YR: validation and investigation. ZR, XN, and YH: funding and supervision. All authors read and agreed to the published version of the manuscript.

## Funding

This research was supported by the Science and Technology Support Program (Grant No. 2020YFS0337) and Fund of Sichuan Province Education Department (Grant No. 18TD0032).

## Conflict of Interest

The authors declare that the research was conducted in the absence of any commercial or financial relationships that could be construed as a potential conflict of interest.

## Publisher's Note

All claims expressed in this article are solely those of the authors and do not necessarily represent those of their affiliated organizations, or those of the publisher, the editors and the reviewers. Any product that may be evaluated in this article, or claim that may be made by its manufacturer, is not guaranteed or endorsed by the publisher.
