# Supplementary figures and images for: Antibacterial Activity of Two Metabolites Isolated From Endophytic Bacteria Bacillus velezensis Ea73 in Ageratina adenophora
Source: Front Microbiol. 2022 May 6;13:860009. doi: 10.3389/fmicb.2022.860009 (PMC9121010; doi:10.3389/fmicb.2022.860009)

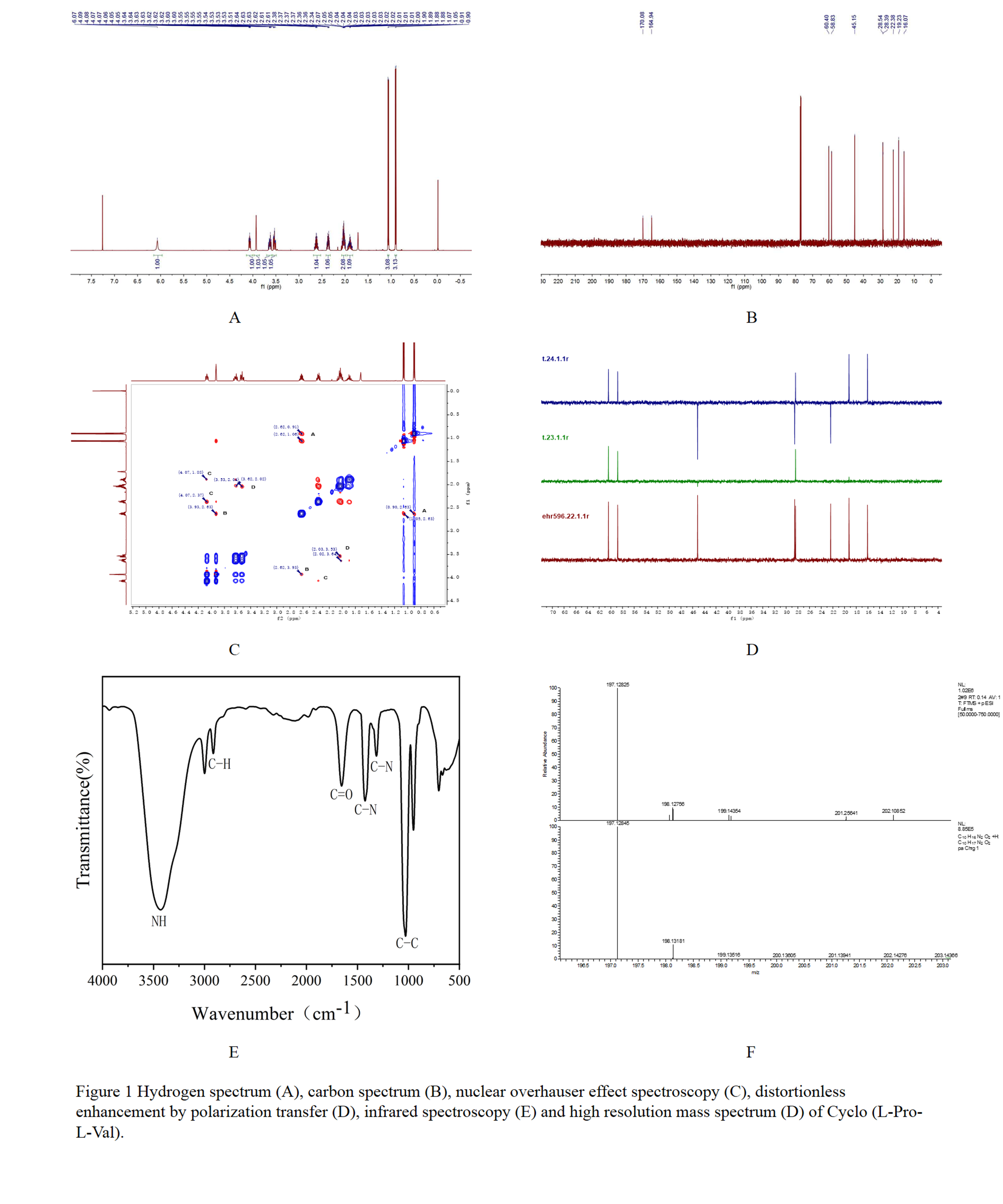

Supplement: Supplementary file 1 [file Image_1.TIF]

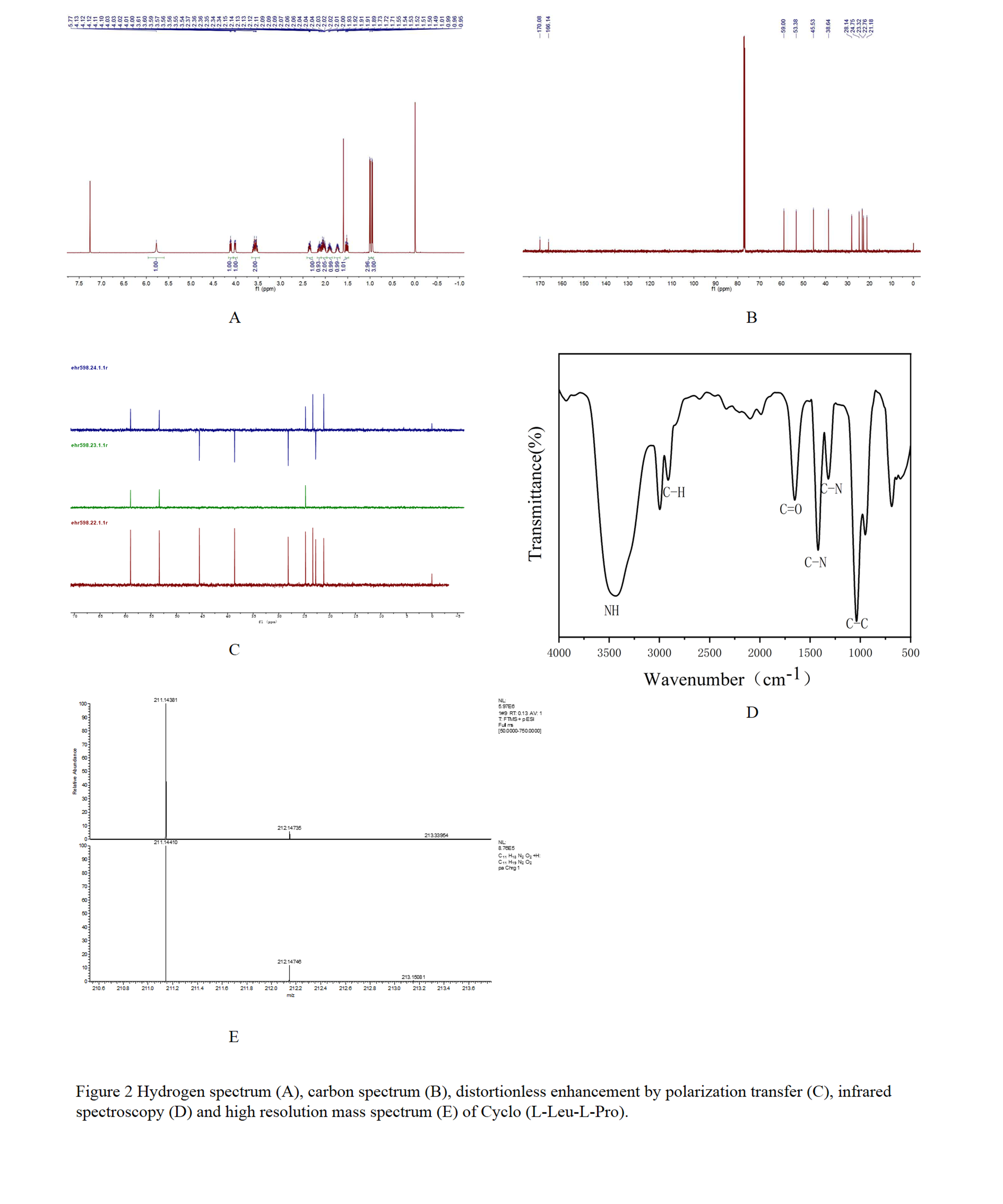

Supplement: Supplementary file 2 [file Image_2.TIF]
